# Pros and Cons of Use of Mitochondria-Targeted Antioxidants

**DOI:** 10.3390/antiox8080316

**Published:** 2019-08-17

**Authors:** Egor Y. Plotnikov, Dmitry B. Zorov

**Affiliations:** 1 A.N. Belozersky Institute of Physico-Chemical Biology, Lomonosov Moscow State University, Moscow 119992, Russia; 2 V.I. Kulakov National Medical Research Center of Obstetrics, Gynecology and Perinatology, Moscow 117997, Russia

**Keywords:** mitochondria, oxidative stress, antioxidants

## Abstract

Mitochondrial targeting is a novel strategy, which addresses pathologies originating from mitochondrial dysfunction. Here, one of the most potent therapeutics arises from the group of mitochondria-targeted antioxidants, which specifically quench mitochondrial reactive oxygen species (ROS). They show very high efficacy in the treatment of a diverse array of pathologies encountered in this Special Issue of *Antioxidants*. However, despite very encouraging results in the use of mitochondria-targeted antioxidants, the mechanistic principle of delivering these agents is, to some extent, counterproductive to the goal of selectively treating a population of damaged mitochondria. The main problem that arises is that injured mitochondria may carry a lower membrane potential when compared with normal ones and as a result, injured mitochondria are capable of taking up less therapeutic antioxidants than healthy mitochondria. Another problem is that the intracellular activity of mitochondrial ROS differs from cytosolic ROS in that they carry specific intracellular functions which are maintained at a delicate equilibrium and which may be disturbed under careless use of antioxidant doses. Consequently, understanding the overall benefit of targeting dysfunctional mitochondria in pathological tissue requires furthering the development of alternative techniques to target mitochondria.

Apart from offering a cure, most types of therapeutic intervention can have numerous adverse side effects, which can be the case with the use of antioxidants. The purpose of using antioxidants is to reduce excessive levels of highly reactive oxidants most of which are represented by reactive oxygen species (ROS). The ROS group consists mainly of unstable radicals (superoxide anion, hydroxyl anion and singlet oxygen, including derivatives thereof) and hydrogen peroxide (reviewed in [1]). All these agents have different oxidizing capacities and potential, possess distinct physical and chemical properties which determine their distinct distribution within hydrophilic and hydrophobic environments and which contribute largely to their substrate specificity. Nevertheless, we must emphasize that ROS are an essential element of cell signaling, regulating vital biochemical processes within the cell, ultimately regulating the processes of division, differentiation, growth, and neutralization or elimination of unwanted cell parts or whole cells [2].

Homeostasis of ROS is one of the mandatory requirements for maintaining the viability of the cellular system [3] and alterations in it are mainly caused by a local insult from a range of chemical, physical and biological factors. As a result, a lesion focus may be formed which emits ROS in excessive levels and becomes a trigger for the emergence of serious pathological conditions often associated with inflammation and a high risk of tissue death [4].

To some extent, the intracellular localization of oxidative stress is the main problem and in a positive manner does, therefore, offer an appropriate therapeutic approach to its elimination. It is fair to say that even in the absence of any oxidative challenge, an organism is a highly heterogeneous system in terms of existing ROS levels. Slowly metabolizing tissues are most often characterized by a low level of endogenous ROS in contrast to tissues with high metabolic activity, such as the heart or brain. Nevertheless, even within these organs, which are composed of a diverse community of cells, there is high heterogeneity in both the levels of ROS and in the activity levels of systems that maintain normal ROS homeostasis [5,6,7]. By definition, high levels of ROS should be observed in the lung tissue, which primarily makes contact with alien antigens from inhaled air at very high partial pressures of oxygen [8,9]. To some extent, this is true for the intestinal tract as well, although it is characterized by possessing low metabolic activity [10].

To suppress the oxidative burst, general antioxidants are therefore quite ubiquitously used. Indeed, general antioxidants are administered orally, iv or intra-muscular, which eventually penetrate into the blood and reach the targeted organ. However, upon using any of these forms of antioxidant administration, healthy tissues other than the targeted organ, which have not experienced oxidative damage, may be non-specifically targeted by the antioxidant [11,12].

Now that it has become common knowledge that mitochondria are one of the main generators of ROS in the cell, it is also accepted that it is through this local “oxidative fire” arising from within the mitochondria that gives rise to pathogenicity [13,14,15]. These arguments serve as a strong ideological basis for the development of the concept in the use of mitochondria-targeted antioxidants. The principle being developed is based on the proposal to use charged substances possessing a number of conjugated double bonds, which permit the charge to be delocalized [16] particularly as the localization of a charge can be the main obstacle in the penetration of ions through phospholipid membranes. This is due to each localized charge in the aqueous phase being surrounded by a shell of oppositely charged ions and water dipoles, which cannot cross the bilayer due to steric restrictions. Delocalization (spreading out) of the primary charge solves the problem of penetration, which leads to the fact that cations possessing delocalized charges can be accumulated in the mitochondrial matrix carrying a negative charge and anions with delocalized charges can accumulate inside the sub-mitochondrial particles carrying a positive charge [17,18]. Without exaggeration, these ions with delocalized charges formed a strong basis for awarding the Nobel prize in 1978 to P. Mitchell, as a result of proving the chemiosmotic hypothesis [19].

However, several decades later, it has become clear that penetrating cations can serve as a locomotive for the transfer of therapeutic agents to the mitochondria. Historically, the first proposition was to use locomotive conjugates made of penetrating cation and antioxidant molecules, primarily quinone derivatives [20]. Once in the mitochondria, reduced quinones (ubiquinone or plastoquinone derivatives) are oxidized, with plastoquinone derivatives being able to receive electrons from the respiratory chain of mitochondria resulting in their reduction, thus making these compounds renewable. Consequently, frenetic activity in developing derivatives of such compounds resumed permitting the mitochondrial “oxidative fire” to be better controlled and even in some cases, completely extinguished [21,22].

The conceptual drive to extinguish oxidative stress in pathological mitochondria was established for a number of pathological processes associated with oxidative stress. For more than two decades of experimental research into mitochondria-targeted antioxidants, a number of impressive beneficial effects in the treatment of numerous pathologies associated with oxidative stress have been demonstrated which offer a wide therapeutic concentration window [23,24,25,26,27,28,29,30,31,32,33,34,35,36,37,38]. For example, in this Special Issue of *Antioxidants* a few successful examples are presented for the treatment of brain trauma [39], age-related macular degeneration [40], light-induced retina damage [41], hearing loss [42], and neonatal sepsis [43]. Related issues such as prevention of oxidative stress-induced mitochondrial dysfunction by mitochondrial uncouplers [44], melatonin [45] and glutathione [46] and physiologic implications of ROS production by a reverse electron transport [47] are also covered in the Special Issue. As we see, the mitochondria-targeting concept is developing exponentially through the exploration of a variety of vehicles and substances targeting the mitochondria with a view to restoring impaired mitochondrial structure and functions.

However, mitochondrial targeting has also faced its fair share of problems and drawbacks. The main problems faced stem from conflicts between theory and practice as the accumulation of mitochondrial-targeted antioxidants should occur in direct proportion to the value of the mitochondrial membrane potential (Δψ), which serves to drive the accumulation of cations in a negatively charged matrix. At the same time, there is evidence that the mitochondrial population is somewhat heterogeneous in terms of Δψ and it is in pathological conditions that this heterogeneity is enhanced due to the appearance of low-potential mitochondria. Consequently, the use of mitochondrial-directed antioxidants meets a paradoxical challenge in that low-potential mitochondria do need quenching for their internal oxidative stress but their uptake of such agents is poor by comparison to healthy mitochondria which do not experience any problem with metabolism.

As a result, upon increasing antioxidant doses to permit the repair of pathological mitochondria (especially toxic hyperpolarized mitochondria), normal mitochondria may also be affected negatively as their levels of ROS may fall below their physiologically acceptable limit. Derived from this, it should also be noted that in the scientific arena, the term “oxidative stress” is often used very broadly while very little mention is made of “reductive stress”, which can be defined by the presence of unacceptably low levels of ROS and which does not allow the normal physiological reactions to occur in the “ROS-world” [4]. This makes it possible to include reductive stress (generalized or local) in the category of no less pathogenic systems than oxidative stress. It is fair to mention that mitochondria-targeted antioxidants will have the maximum effect on hyperpolarized mitochondria, which are also likely to be toxic to cells. Redefining the consequences of ROS damage based on them occurring through simultaneous variations in the levels of oxidative- or reductive-stresses allows us to therefore define the pathologies of diseases arising from them with greater accuracy as well. 

The problem of heterogeneity of the mitochondrial population in the cell is directly related to the above-mentioned heterogeneity of ROS generation. As of yet, no regulatory mechanisms have been defined that permit the discrimination between ROS species as being “good ROS” or “bad ROS” as the detrimental effects of ROS are believed to be defined by their origins and what levels they appear to be present in. There are many examples of mitochondrial (rather than cytosolic) ROS that participate in a number of specific molecular signaling events and consequently, the ablation of mitochondrial or cytoplasmic ROS below physiological thresholds can lead to deregulation or loss of specific signaling cascades.

Thus, with all the obvious benefits of mitochondria-targeted antioxidants resulting from the diverse origins of ROS distribution in the organism, organs, and cells, we have to be mindful of dosage, timing, and modes of exposure for different pathologies since the heterogeneity of the mitochondrial population has to be a central consideration. Alternatively, formulating an approach which does not exploit the mitochondrial membrane potential to drive antioxidant accumulation by specifically targeting unique mitochondrial components (such as oxidized cardiolipin) may offer a more specific approach in treating a damaged population of mitochondria.
